# Supplementary material for: Divergent patterns of cognitive decline in preclinical Alzheimer’s disease: implications for secondary prevention trials
Source: medRxiv. 2026 Jan 21:2025.12.15.25342305. Originally published 2025 Dec 17. Preprint. [Version 2] doi: 10.64898/2025.12.15.25342305 (PMC12723760; doi:10.64898/2025.12.15.25342305)
Supplement: 1 [file NIHPP2025.12.15.25342305v2-supplement-1.pdf]

medRxiv preprint doi: https://doi.org/10.64898/2025.12.15.25342305; this version posted January 21, 2026. The copyright holder for this preprint
(which was not certified by peer review) is the author/funder, who has granted medRxiv a license to display the preprint in perpetuity.
It is made available under a CC-BY 4.0 International license .

Supplementary Material

medRxiv preprint doi: https://doi.org/10.64898/2025.12.15.25342305; this version posted January 21, 2026. The copyright holder for this preprint (which was not certified by peer review) is the author/funder, who has granted medRxiv a license to display the preprint in perpetuity.
It is made available under a CC-BY 4.0 International license .

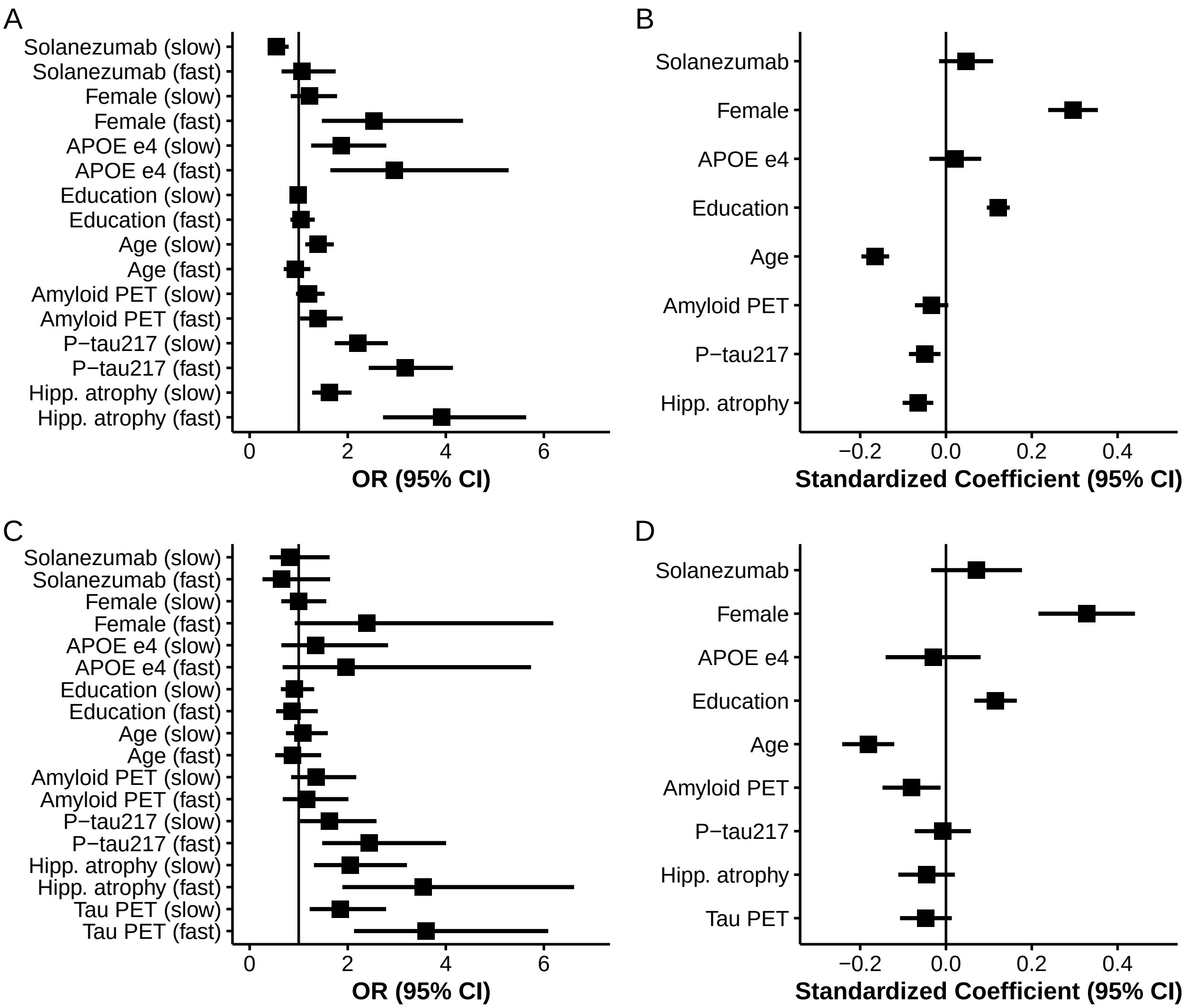

Supplementary Figure S1: **Association of baseline predictors with latent class membership and longitudinal cognitive decline in models with and without tau PET.** Panels A and B display results from the latent class mixed model (LCMM) excluding tau PET; panels C and D show corresponding results from the model including tau PET as an additional predictor. (A, C) Odds ratios (95% CIs) from the LCMM class-membership submodel represent the relative likelihood of belonging to the slow- or fast-declining classes relative to stable class. (B, D) Standardized coefficients (95% CIs) from the LCMM longitudinal submodel show associations between baseline predictors and rate of change in the Preclinical Alzheimer Cognitive Composite (PACC). All continuous predictors and the PACC outcome were standardized (z-scored) before analysis to enable comparison of effect magnitudes. Higher PACC scores indicate better cognitive performance; thus, larger positive coefficients correspond to slower decline or better preservation of cognition over time.

medRxiv preprint doi: https://doi.org/10.64898/2025.12.15.25342305; this version posted January 21, 2026. The copyright holder for this preprint (which was not certified by peer review) is the author/funder, who has granted medRxiv a license to display the preprint in perpetuity.
It is made available under a CC-BY 4.0 International license .

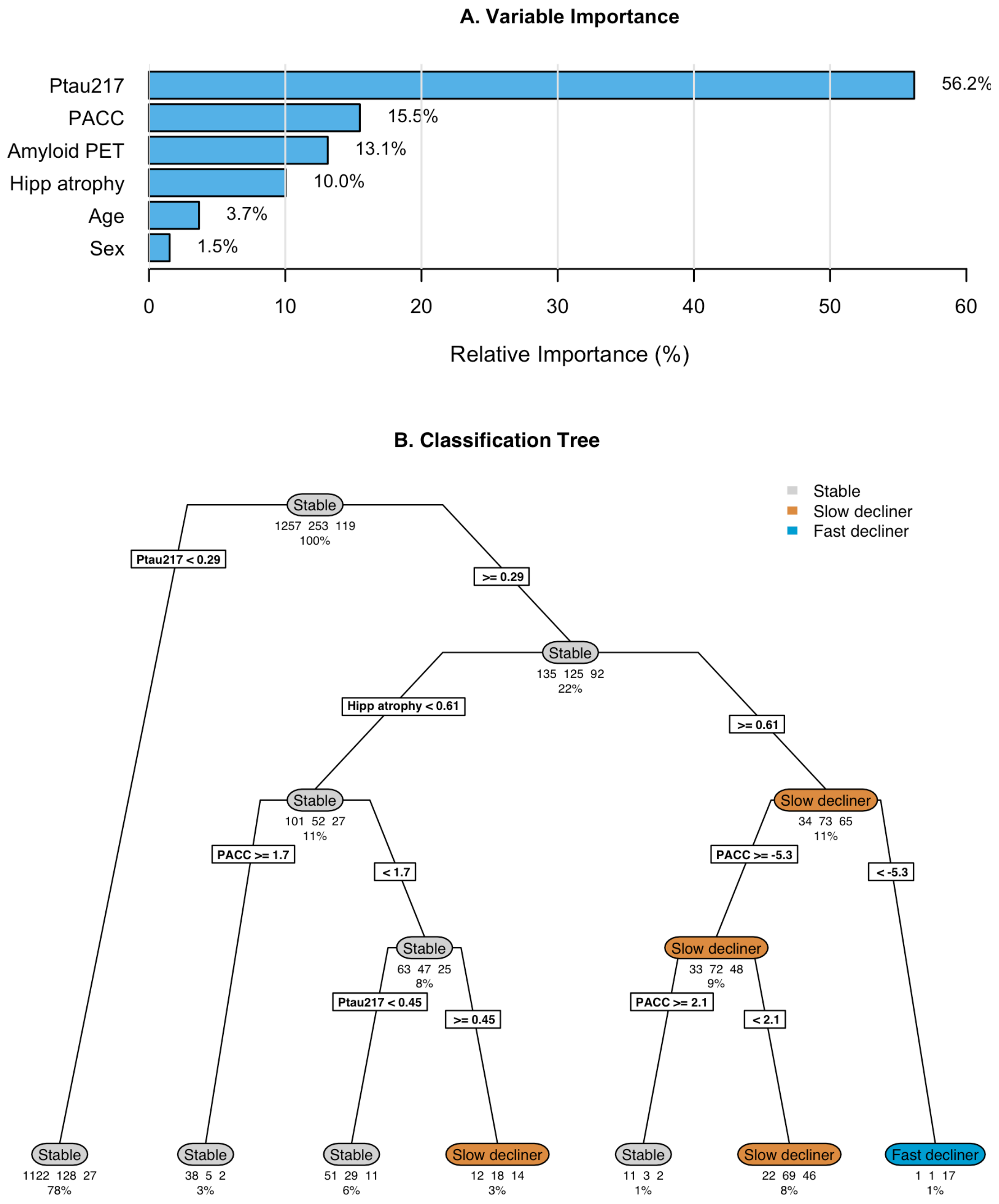

Supplementary Figure S2: **Classification tree analysis for latent class assignment from Base Model.** Panel (A) shows relative importance of baseline predictors for class separation. Panel (B) shows classification tree showing binary decision rules for assigning subjects to latent classes based on predictors. Terminal nodes show predicted class, number of subjects, and percentage of total sample. The optimal tree complexity was determined using 10-fold cross-validation with the 1-standard error rule, resulting in a tree with 6 splits (mean balanced accuracy = 0.63, standard error 0.007)

medRxiv preprint doi: https://doi.org/10.64898/2025.12.15.25342305; this version posted January 21, 2026. The copyright holder for this preprint (which was not certified by peer review) is the author/funder, who has granted medRxiv a license to display the preprint in perpetuity.
It is made available under a CC-BY 4.0 International license .

	Stable (N=312)	Slow decliner (N=73)	Fast decliner (N=42)	Total (N=427)	p value
Group					0.004
- LEARN	52 (16.7%)	3 (4.1%)	0 (0.0%)	55 (12.9%)	
- Placebo	131 (42.0%)	34 (46.6%)	23 (54.8%)	188 (44.0%)	
- Solanezumab	129 (41.3%)	36 (49.3%)	19 (45.2%)	184 (43.1%)	
Age, mean (SD), y	71.15 (4.51)	73.74 (4.83)	72.80 (5.65)	71.76 (4.78)	$<$ 0.001
Sex, No. (%)					0.009
- Male	126 (40.4%)	40 (54.8%)	11 (26.2%)	177 (41.5%)	
- Female	186 (59.6%)	33 (45.2%)	31 (73.8%)	250 (58.5%)	
Race, No. (%)					0.426
- N missing	3	0	1	4	
- Am. Indian or Alaska Native	1 (0.3%)	0 (0.0%)	0 (0.0%)	1 (0.2%)	
- Asian	12 (3.9%)	2 (2.7%)	4 (9.8%)	18 (4.3%)	
- Native Hawaiian or Other PI	0 (0.0%)	0 (0.0%)	0 (0.0%)	0 (0.0%)	
- Black or African Am.	7 (2.3%)	4 (5.5%)	0 (0.0%)	11 (2.6%)	
- White	287 (92.9%)	67 (91.8%)	37 (90.2%)	391 (92.4%)	
- Unknown or Not Reported	2 (0.6%)	0 (0.0%)	0 (0.0%)	2 (0.5%)	
Ethnicity, No. (%)					0.842
- Hispanic or Latino	5 (1.6%)	1 (1.4%)	1 (2.4%)	7 (1.6%)	
- Not Hispanic or Latino	302 (96.8%)	70 (95.9%)	41 (97.6%)	413 (96.7%)	
- Unknown or Not reported	5 (1.6%)	2 (2.7%)	0 (0.0%)	7 (1.6%)	
Education, mean (SD), y	16.24 (2.89)	16.16 (2.66)	16.05 (2.70)	16.21 (2.83)	0.904
APOE 4, No. (%)					$<$ 0.001
- non-carrier	166 (53.2%)	22 (30.1%)	11 (26.2%)	199 (46.6%)	
- carrier	146 (46.8%)	51 (69.9%)	31 (73.8%)	228 (53.4%)	
PACC, mean (SD)	0.51 (2.48)	-1.20 (2.57)	-2.57 (3.20)	-0.09 (2.77)	$<$ 0.001
Amyloid PET, mean (SD), CL	47.04 (30.54)	77.33 (38.73)	86.23 (34.78)	56.07 (35.74)	$<$ 0.001
P-tau217, mean (SD), U/ml	0.22 (0.10)	0.33 (0.17)	0.43 (0.20)	0.26 (0.15)	$<$ 0.001
Hipp. atrophy, mean (SD), z-score	0.03 (0.83)	0.80 (0.85)	0.96 (0.79)	0.25 (0.91)	$<$ 0.001
Tau PET, mean (SD), SUVr	1.08 (0.07)	1.13 (0.08)	1.23 (0.13)	1.10 (0.09)	$<$ 0.001
CDR Prog., No. (%)					$<$ 0.001
- CDR Non-progressor	217 (69.6%)	18 (24.7%)	2 (4.8%)	237 (55.5%)	
- CDR Progressor	95 (30.4%)	55 (75.3%)	40 (95.2%)	190 (44.5%)	

Supplementary Table S1: **Baseline Demographic, Clinical, and Biomarker Characteristics by Latent Class of Cognitive Decline with Tau PET.** Baseline characteristics of participants classified by the latent class mixed model (LCMM) of Preclinical Alzheimer Cognitive Composite (PACC) trajectories. Classes represent distinct longitudinal cognitive patterns: stable, slow decliner, and fast decliner. Continuous variables are presented as mean (SD); categorical variables as No. (%). **Abbreviations:** APOE = apolipoprotein E; PET = positron emission tomography; P-tau217 = plasma phosphorylated tau 217; SUVr = standardized uptake value ratio; U/mL = arbitrary units proportional to assay signal; Am. = American; PI = Pacific Islander; CDR = Clinical Dementia Rating; Prog. = Progressor. **Footnotes:** Amyloid PET and tau PET SUVr values represent mean cortical uptake relative to cerebellar reference region. Hippocampal atrophy are residualized for intracranial volume and z-scored. Education reported in years of formal schooling. CDR Progressors were observed to have a CDR Global score greater than zero at two consecutive visits, or their last visit.

medRxiv preprint doi: https://doi.org/10.64898/2025.12.15.25342305; this version posted January 21, 2026. The copyright holder for this preprint (which was not certified by peer review) is the author/funder, who has granted medRxiv a license to display the preprint in perpetuity.
It is made available under a CC-BY 4.0 International license .

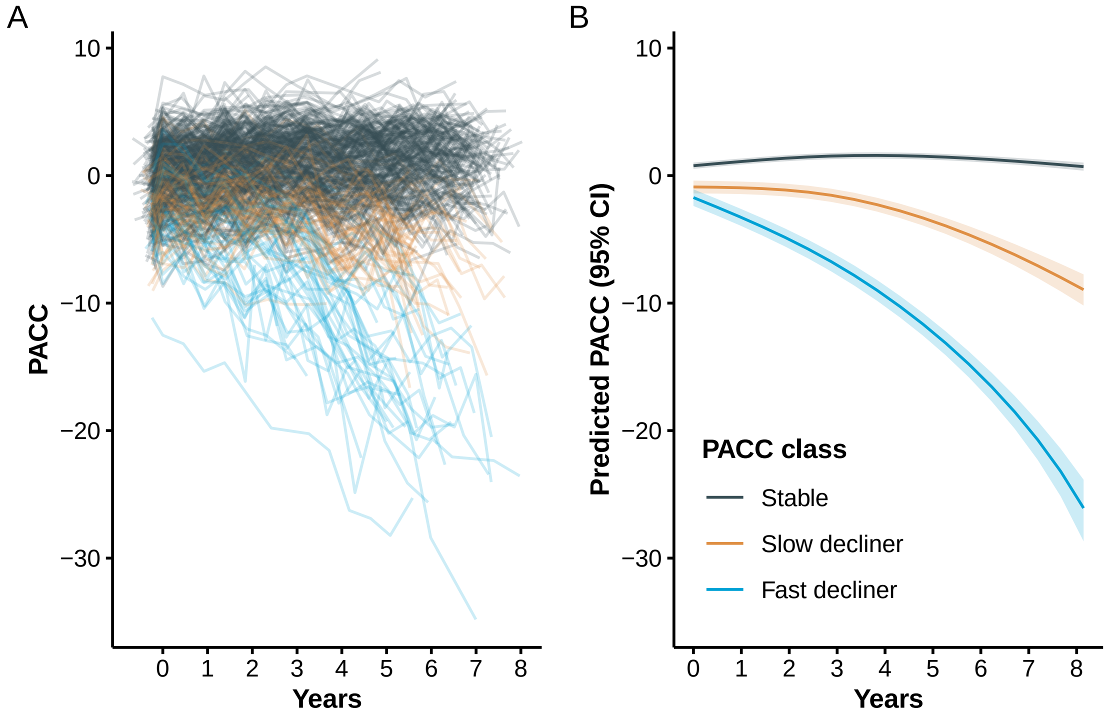

Supplementary Figure S3: **Individual and Mean PACC Trajectories by Latent Class Model with Tau PET.** Left panel (A): Spaghetti plot of individual participant trajectories on the Preclinical Alzheimer Cognitive Composite (PACC), colored by latent class derived from the latent class mixed model (LCMM) which included tau PET as a predictor. Each line represents one participant's observed scores over time. Right panel (B): Estimated mean PACC trajectories for each latent class, with shaded regions indicating 95% confidence intervals. Higher PACC scores indicate better cognitive performance. **Abbreviations:** PET = positron emission tomography.

medRxiv preprint doi: https://doi.org/10.64898/2025.12.15.25342305; this version posted January 21, 2026. The copyright holder for this preprint (which was not certified by peer review) is the author/funder, who has granted medRxiv a license to display the preprint in perpetuity.
It is made available under a CC-BY 4.0 International license .

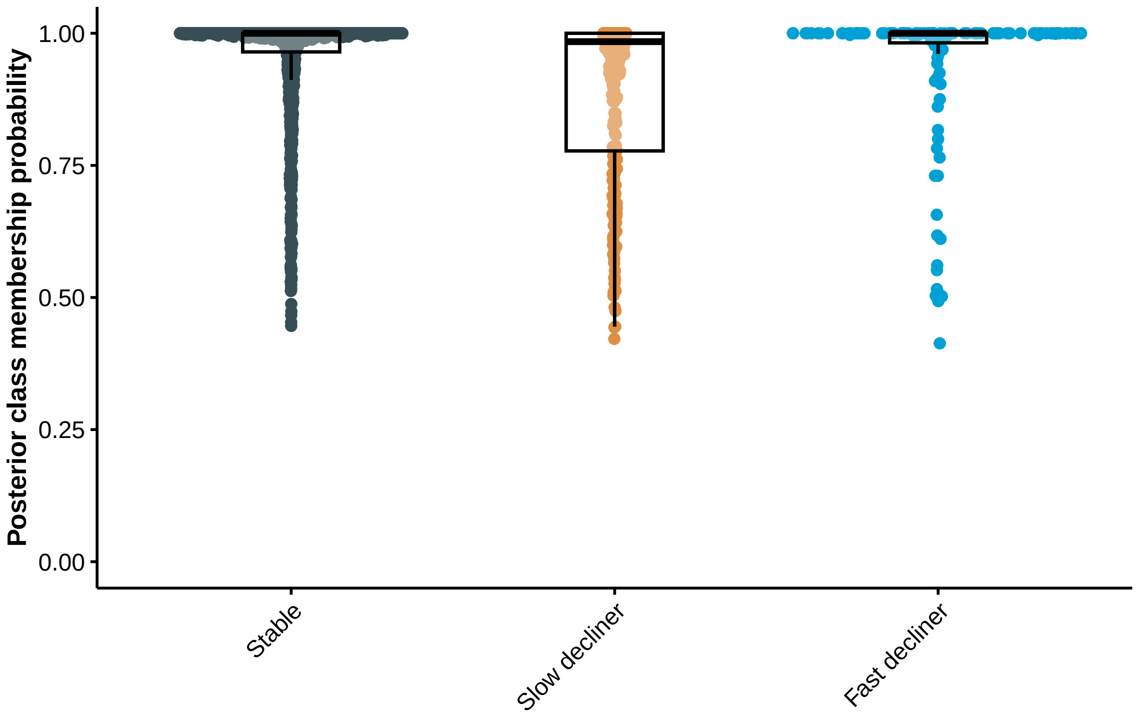

Supplementary Figure S4: **Distribution of posterior class membership probabilities by assigned latent class, indicating model confidence in class assignment.** This plot shows the degree of certainty in class assignments, a good indicator of model reliability. Mean posterior probabilities were `{r}pp_sum[[1,2]]`, `{r}pp_sum[[2,2]]`, and `{r}pp_sum[[3,2]]` for non-, slow-, and fast decliners. With most participants having high posterior probabilities (> 0.8) for their assigned class, class separation is strong and predictive discrimination is good.

medRxiv preprint doi: https://doi.org/10.64898/2025.12.15.25342305; this version posted January 21, 2026. The copyright holder for this preprint (which was not certified by peer review) is the author/funder, who has granted medRxiv a license to display the preprint in perpetuity.
It is made available under a CC-BY 4.0 International license .

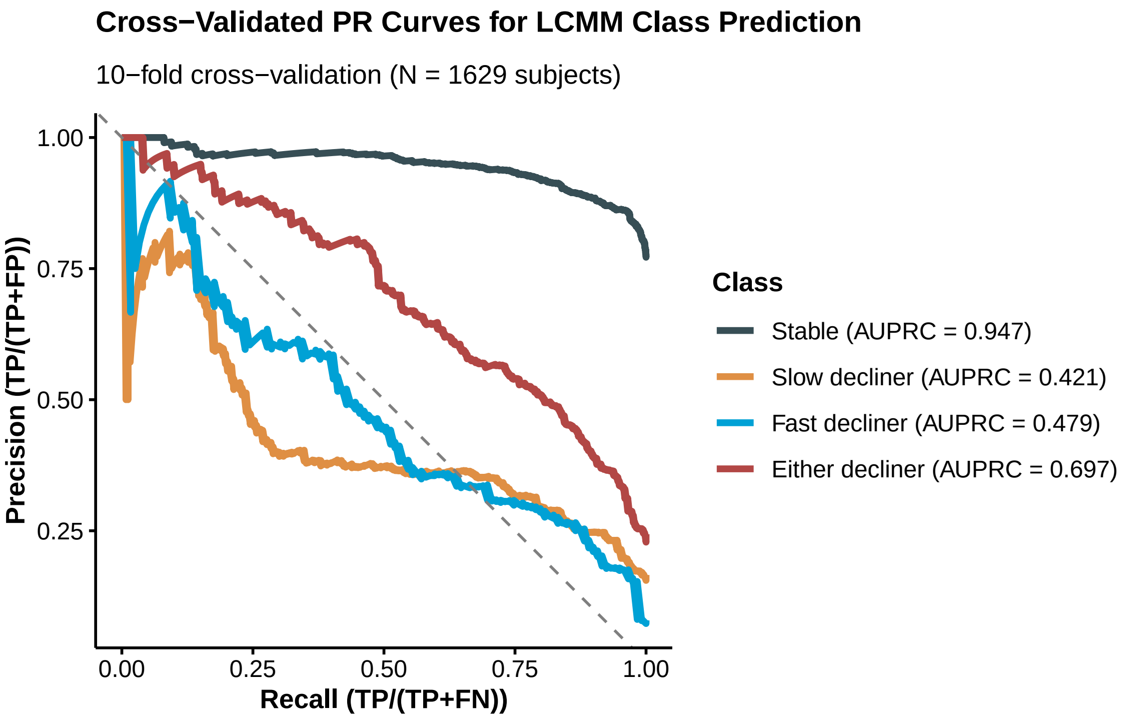

Supplementary Figure S5: **Cross-validated Precision-Recall Curves for Latent Class Membership Model Without Tau PET.** Precision-recall curves summarizing 10-fold cross-validated classification performance of the latent class membership submodel based on baseline demographics and biomarkers, excluding tau PET. Curves depict the ability to distinguish the given class from the other two. The area under the precision-recall curve (AUPRC) quantifies overall predictive performance, emphasizing recall (or sensitivity, TP/(TP+FN)) and precision (TP/(TP+FP)) for less prevalent classes. **Abbreviations:** AUPRC = area under the precision-recall curve; TP = true positive rate; FN = false negative rate; FP = false positive rate.

medRxiv preprint doi: https://doi.org/10.64898/2025.12.15.25342305; this version posted January 21, 2026. The copyright holder for this preprint
(which was not certified by peer review) is the author/funder, who has granted medRxiv a license to display the preprint in perpetuity.
It is made available under a CC-BY 4.0 International license .

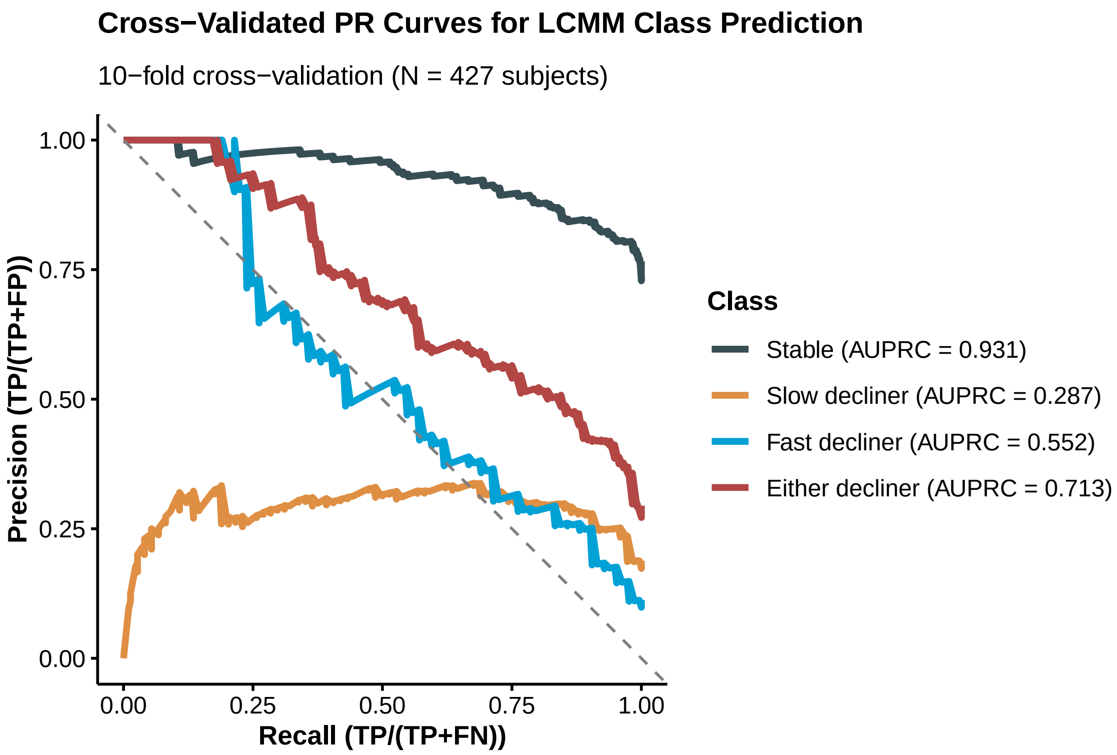

Supplementary Figure S6: **Cross-validated Precision-Recall Curves for Latent Class Membership Model With Tau PET.** Precision-recall curves summarizing 10-fold cross-validated classification performance of the latent class membership submodel based on baseline demographics and biomarkers, excluding tau PET. Curves depict the ability to distinguish the given class from the other two. The area under the precision-recall curve (AUPRC) quantifies overall predictive performance, emphasizing recall (or sensitivity, TP/(TP+FN)) and precision (TP/(TP+FP)) for less prevalent classes. **Abbreviations:** AUPRC = area under the precision-recall curve; TP = true positive rate; FN = false negative rate; FP = false positive rate.

medRxiv preprint doi: https://doi.org/10.64898/2025.12.15.25342305; this version posted January 21, 2026. The copyright holder for this preprint (which was not certified by peer review) is the author/funder, who has granted medRxiv a license to display the preprint in perpetuity.
It is made available under a CC-BY 4.0 International license .

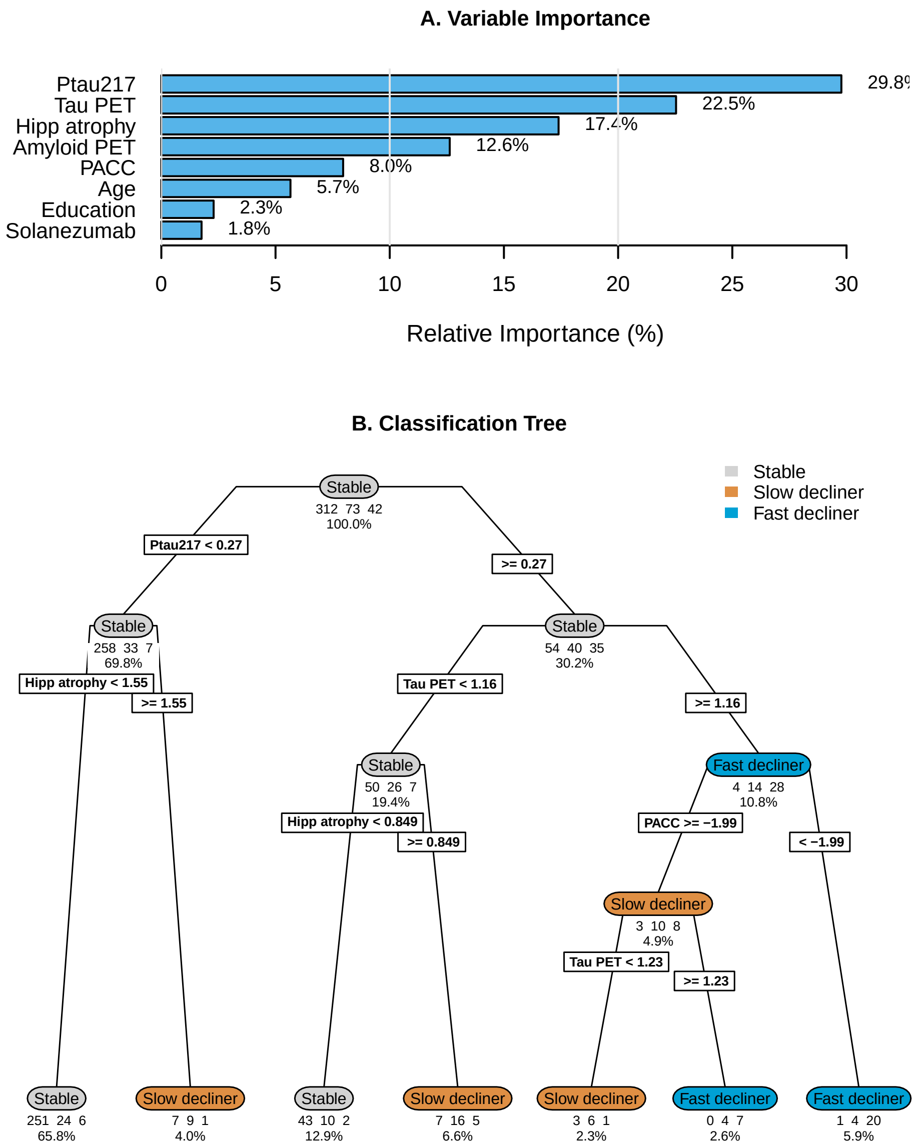

Supplementary Figure S7: **Classification tree analysis for latent class assignment from Tau PET Model.** (A) Relative importance of baseline characteristics for class separation. (B) Classification tree showing binary decision rules for assigning subjects to latent classes based on baseline characteristics. Terminal nodes show predicted class, number of subjects, and percentage of total sample. The optimal tree complexity was determined using 10-fold cross-validation with the 1-standard error rule, resulting in a tree with 6 splits (mean balanced accuracy = 0.70, standard error 0.011).

medRxiv preprint doi: https://doi.org/10.64898/2025.12.15.25342305; this version posted January 21, 2026. The copyright holder for this preprint (which was not certified by peer review) is the author/funder, who has granted medRxiv a license to display the preprint in perpetuity.
It is made available under a CC-BY 4.0 International license .

Group	Follow-up (yrs)	Mean PACC untreated	Standard deviation	Delta (%)	Delta (PACC points)	Power (%)
Decliners	2	-2.80	3.42	20	0.79	93.13
Decliners	4	-5.79	5.00	20	1.40	97.66
Stable Amyloid+	2	0.88	2.19	100	0.26	43.88
Stable Amyloid+	4	0.96	2.34	100	0.24	29.54

Supplementary Table S2: **Power for latent class-specific clinical trials.** Effect size estimates were derived from longitudinal models using natural cubic splines (two degrees of freedom) applied to Preclinical Alzheimer Cognitive Composite (PACC) scores among amyloid-positive A4 participants in (a) the stable class and (b) the two decliner classes. The table reports mean PACC and residual variance at 2 and 4 years, representing expected control group trajectories for two potential trial durations. Power was approximated using two-sample t-test calculations assuming 500 participants per group, with 10% attrition at 2 years and 20% at 4 years. Effect size is expressed both as absolute PACC change and as a percentage of the maximum possible improvement, defined as the mean PACC among stable amyloid-negative LEARN participants at the corresponding time point.
